# TEN-YEAR follow-up of treatment with zygomatic implants and replacement of hybrid dental prosthesis by ceramic teeth: A case report

**DOI:** 10.1016/j.amsu.2019.11.022

**Published:** 2019-12-05

**Authors:** Paulo H.T. Almeida, Sergio H. Cacciacane, Ayrton Arcazas Junior

**Affiliations:** Department of Dental Surgery, São Leopoldo Mandic Institute and Dental Research Center, Campinas, SP, Brazil

**Keywords:** Zygomatic implants, Atrophic maxilla, Local anesthesia, Patient satisfaction, Follow-up

## Abstract

The aim of this case report was to show the 10-year follow-up of a zygomatic implant supported rehabilitation treatment to replace the hybrid dental prosthesis with resin teeth - one by one - with ceramic teeth. The complications that occurred were described right from the time when the first implant supported prosthesis with immediate loading was placed, through the fabrication of a personalized dental prosthesis with twelve ceramic crowns, with a view to achieving esthetic excellence and restoring the patient's self-esteem. It was concluded that all patients with zygomatic implants must participate in a preventive maintenance program to assure the predictability of this type of treatment.

## Highlights

• Surgery may occur in the private clinic with local anesthesia and oral sedation.
• Treatments have high success rates, improvement in quality of life.
• All patients must participate in a preventive maintenance program.

## 1 Introduction

Complete or partial zygomatic implant supported prosthetic rehabilitation has become a safe and predictable treatment over the last 30 years [1]. Initially, zygomatic implants were placed with the original technique, leading to various problems because the implant head was palatinized [2]. Soft tissue inflammation around the microunit abutments, sinusitis, phonetic and cleaning problems, hybrid prostheses with enormous palatal cantilevers were common occurrences [1,3]. Nevertheless, the success rates were always high, above 97% [[4], [5], [6]].

The advent of this approach to treatment began by means of variations in the surgical techniques and the ZAGA philosophy, in which the zygomatic implants are place according to the patient's facial anatomy [2]. Their trajectory could be intra- or extra-maxillary sinus, with the implant head localized closer to the crest of the alveolar ridge [2,7]. The new implant designs also contributed to improving the contact with the peri-implant soft tissue and minimize possible sinus treatments when the trajectory of the implant was within the maxillary sinus and at least had a smooth middle third, without threads [7,8].

The aim of this case report was to show the 10-year follow-up of a zygomatic implant supported rehabilitation treatment and replacement of the hybrid dental prosthesis with resin teeth - one by one - with ceramic teeth.

## 2 Case report

**Fig. 1:**
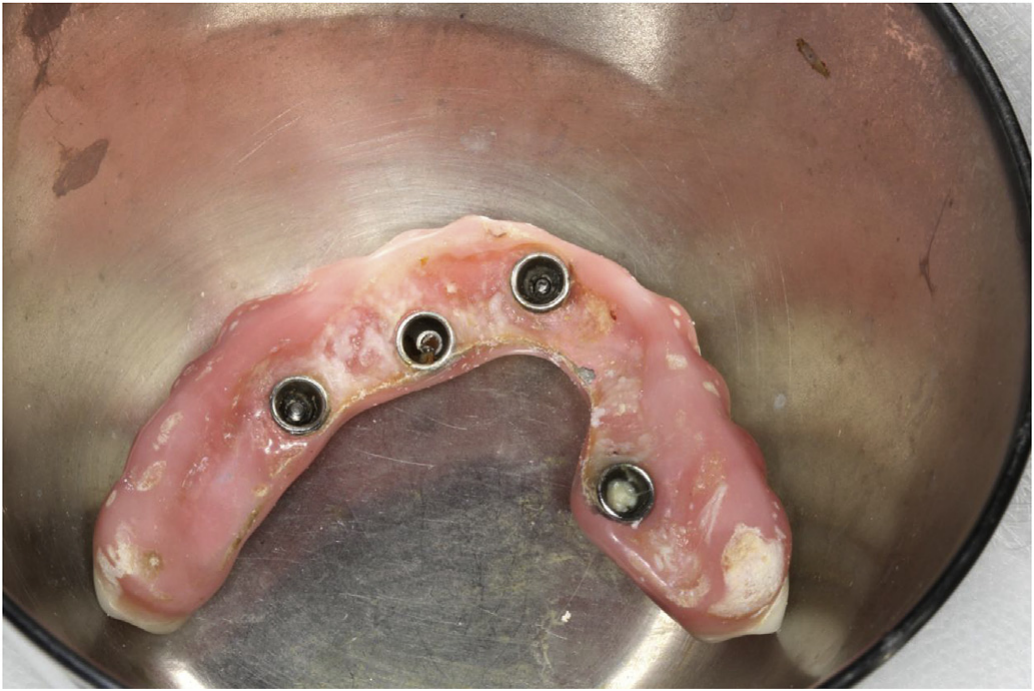
Hybrid denture with resin teeth showing salivary calculus and poor cleaning.

**Fig. 2:**
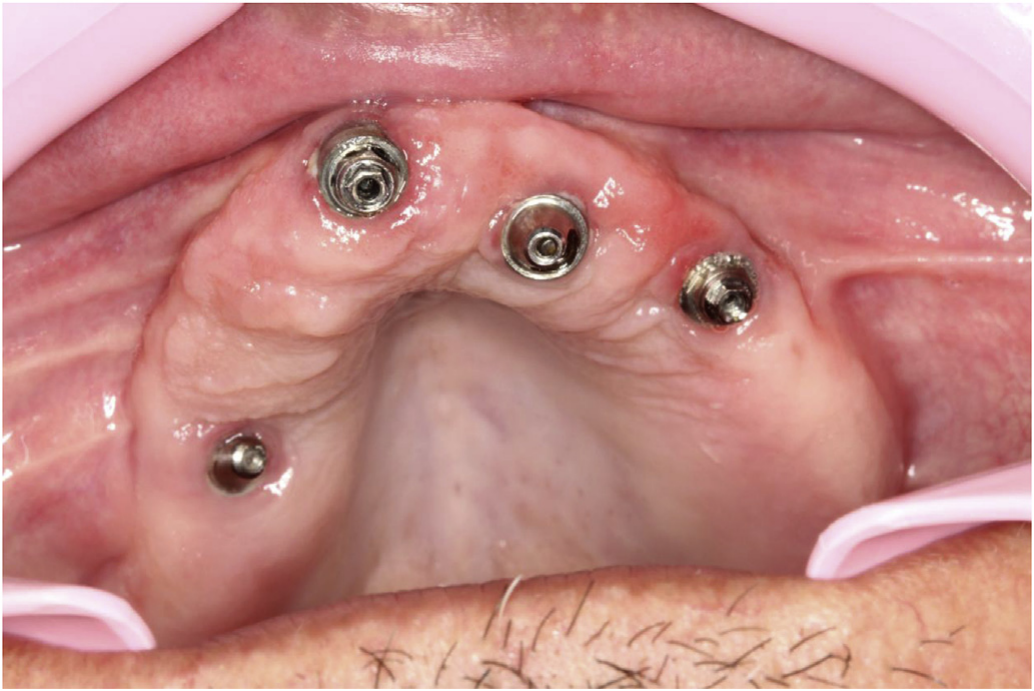
Mucositis.

**Fig. 3:**
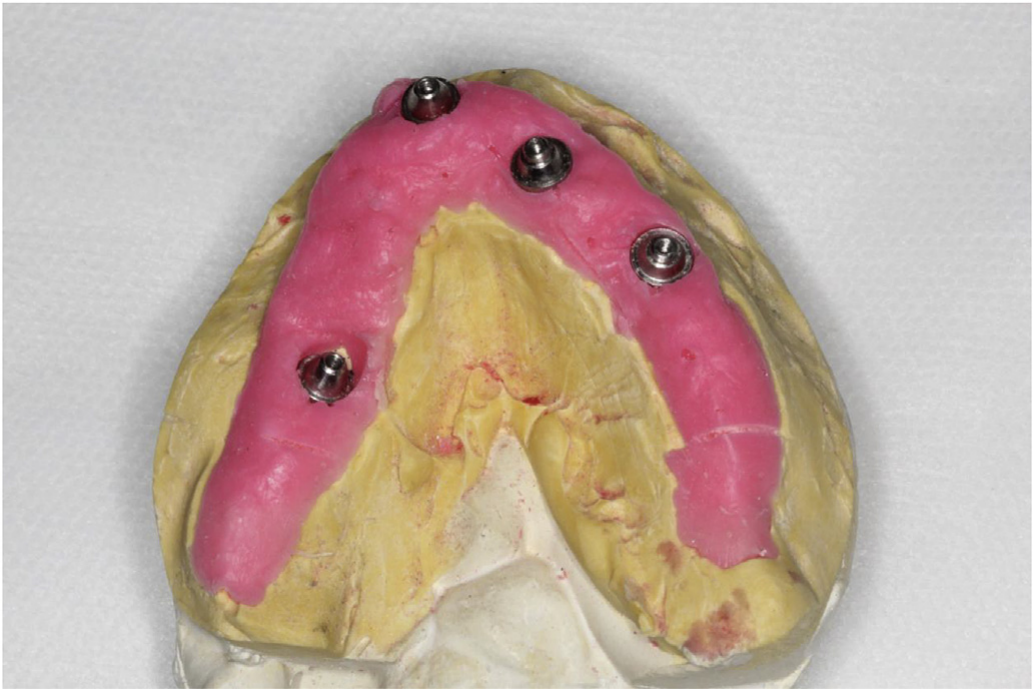
Maxillary model for fabricating the occlusal orientation plane.

**Fig. 4:**
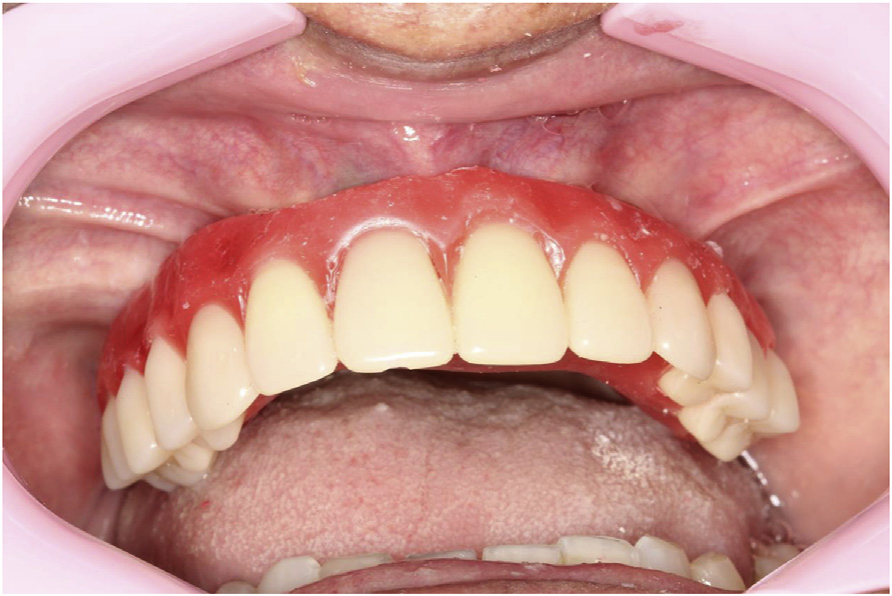
Try-in of resin teeth.

**Fig. 5:**
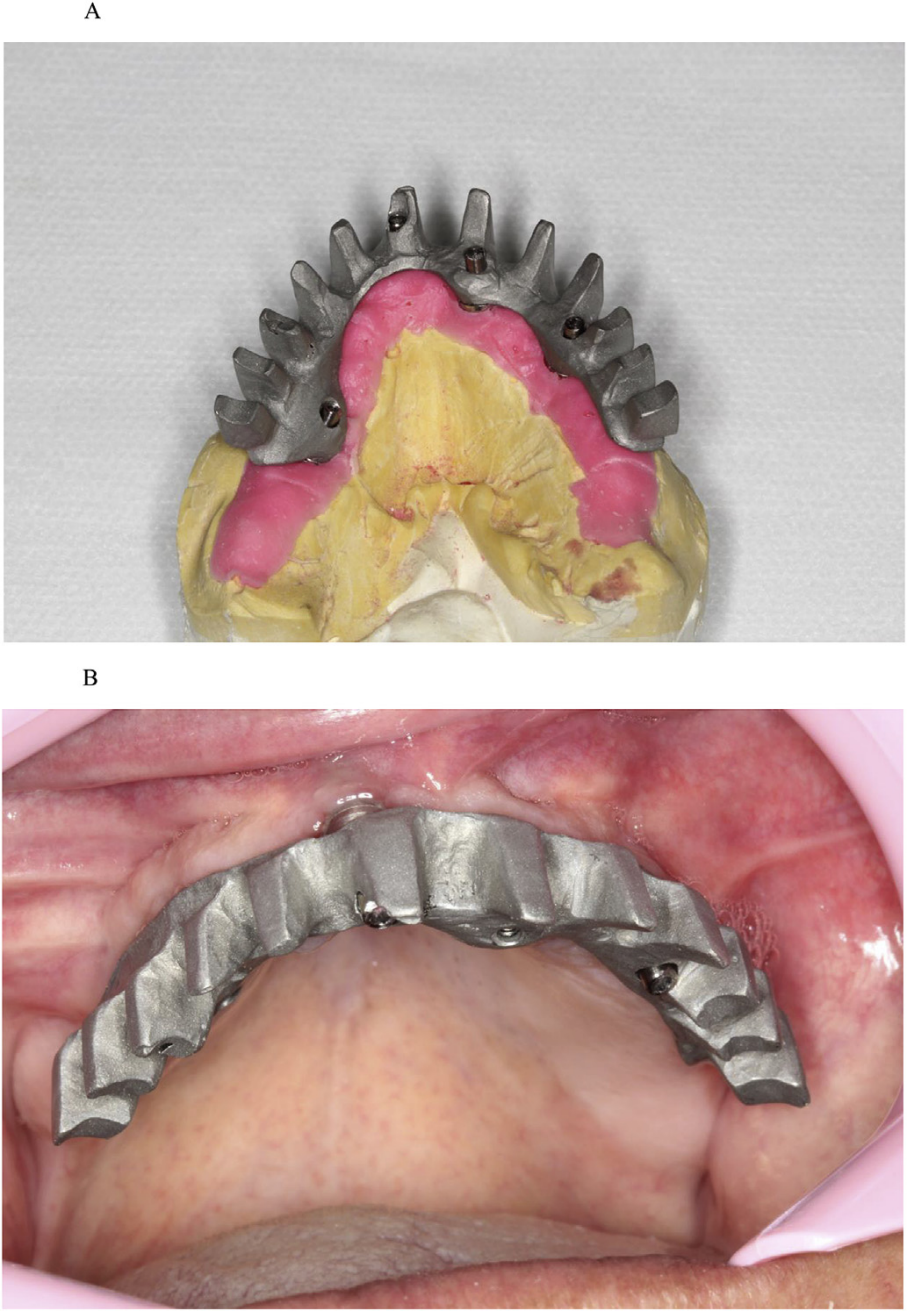
(A). Bar. (B). Screw-retained bar.

**Fig. 6:**
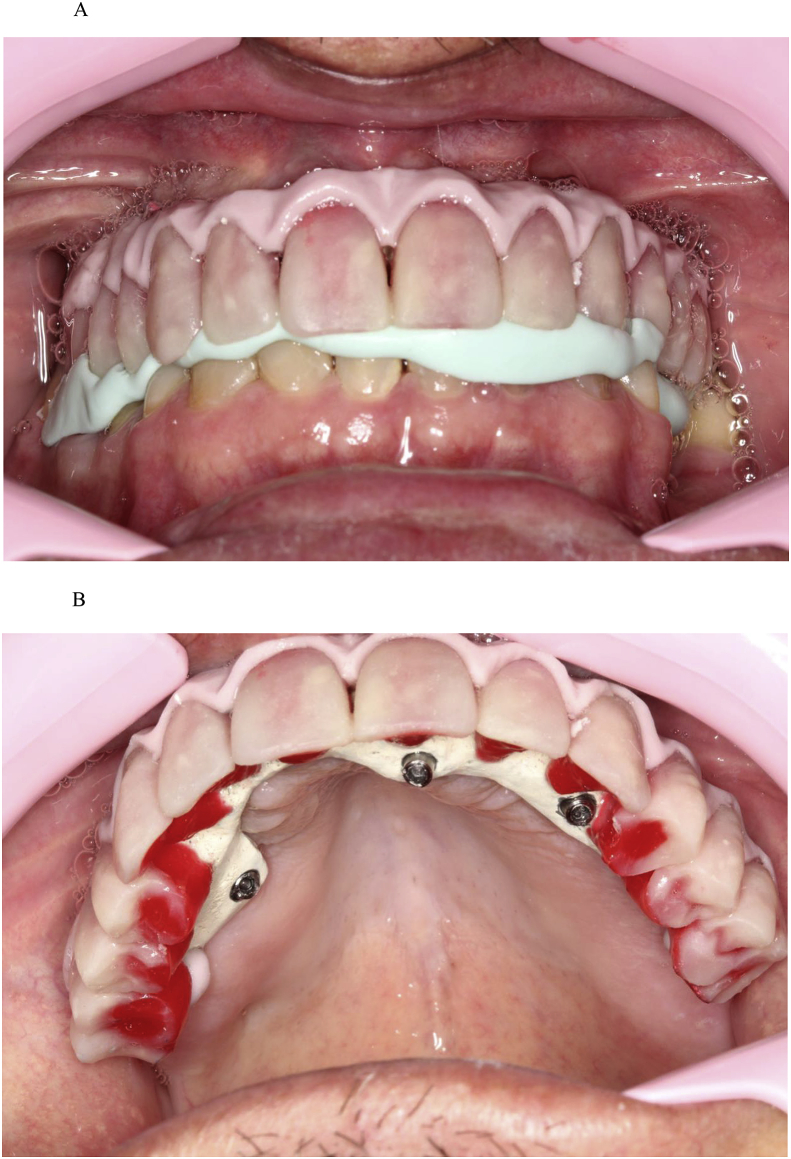
(A). Esthetic try-in and checking occlusion. (B). Teeth made of wax on the acrylic resin casings.

**Fig. 7:**
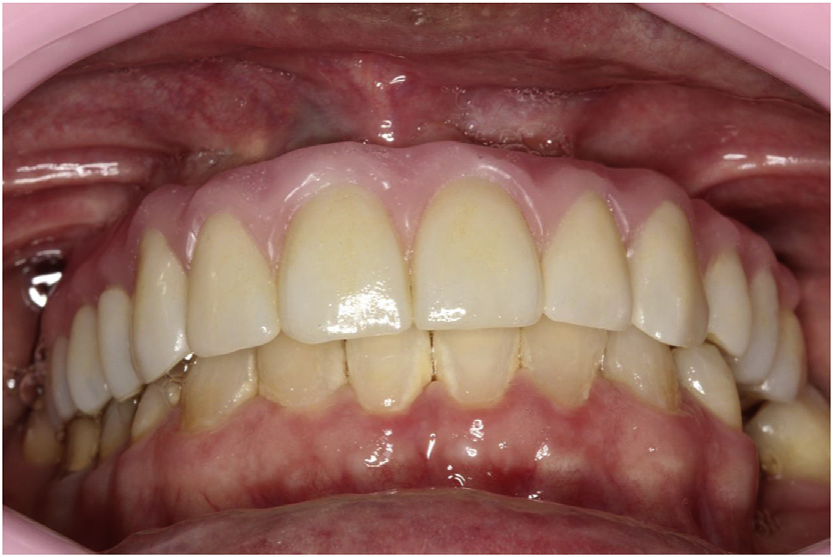
Try-in of teeth - single ceramic crowns.

**Fig. 8:**
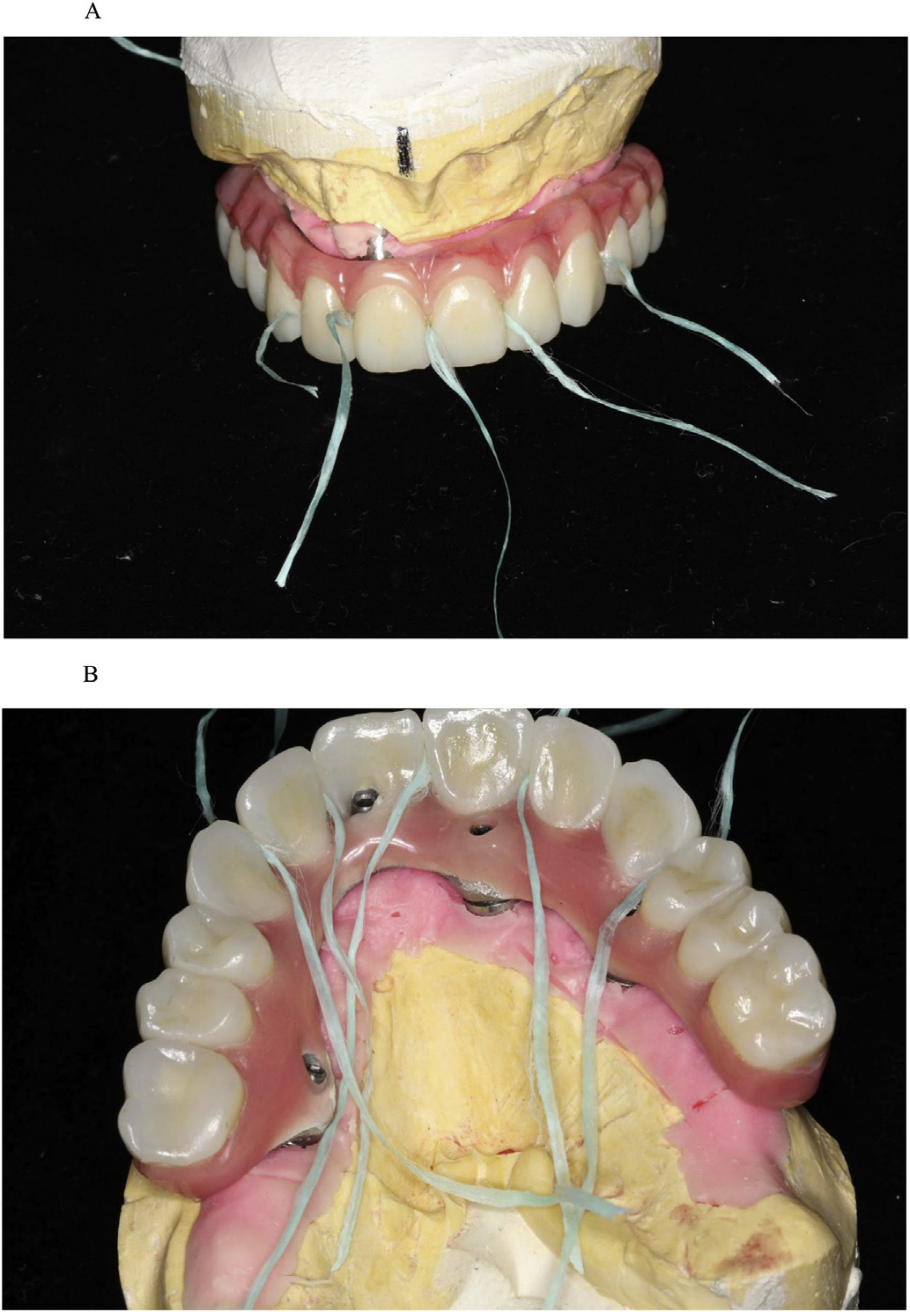
(A). Dental floss between the cemented single crowns. (B). Dental floss between the cemented single crowns.

**Fig. 9:**
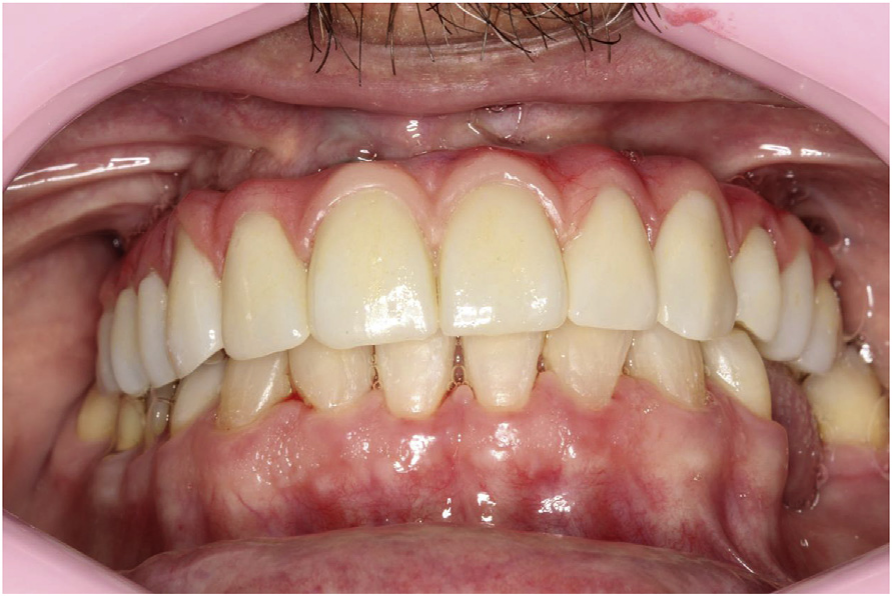
Ceramic tooth by tooth hybrid denture screw-retained on the microunit abutments.

**Fig. 10:**
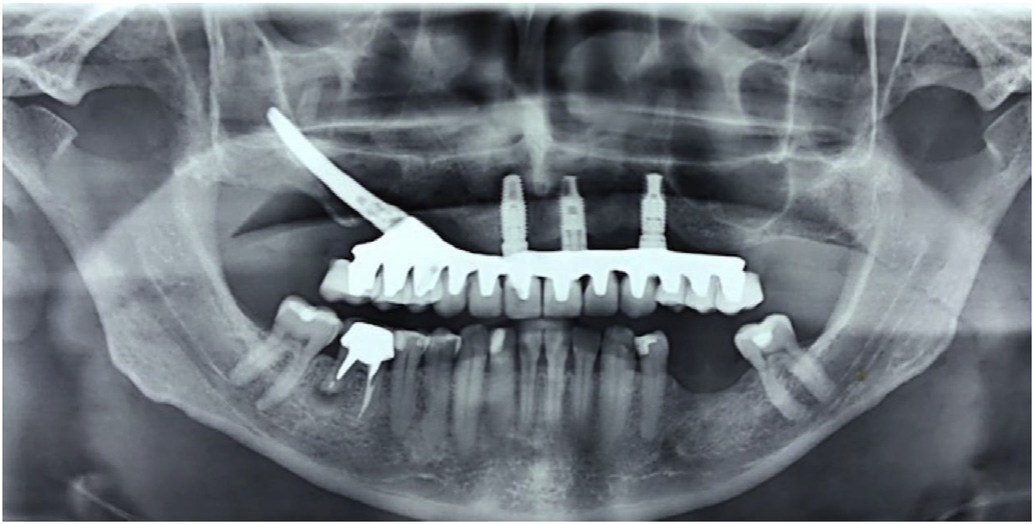
Final radiograph.

The patient, a 48-year-old man, wearer of a removable partial denture in the maxillary arch, presented to a private clinic in October 2009, with the intention of undergoing rehabilitation of the maxilla with a fixed implant supported dental prosthesis. After analyzing the radiographic exams, the treatment options were presented to the patient, who chose the placement of standard dental implants by the all-on-4 technique [9]; on which there would be a hybrid denture with resin teeth. After reverse planning and fabrication of the surgical guide, the surgery was scheduled for February 2010. After extraction of all the remaining teeth in the maxilla, 3 conventional internal hexagon implants (Conexão Sistemas de Prótese Ltda, São Paulo, Brazil) were placed. These were Conect Conico 4.3 × 11.5 mm in the region of tooth 24, with insertion torque of 40 N; Conect AR 3.75 × 13 mm in the region of tooth 21. with torque of 45 N, and Conect Conico 5.0 × 13 mm in the region of tooth 11, with torque of 80 N. Because teeth 14 and 15 were ankylosed, the vestibular bone wall in their region was lost after extraction, making it unfeasible to place standard dental implants, even if they were inclined/tilted. At this time, after obtaining the patient's consent, we opted for the placement of an internal hexagon zygomatic implant (Conexão Sistemas de Prótese Ltda, São Paulo, Brazil) Zigomax 4.0 × 40 mm and insertion torque of 80 N. Straight microunit abutments (Conexão Sistemas de Prótese Ltda, São Paulo, Brazil), were placed with a 2mm band in the region of teeth 24 and 11; at an angle of 17° with a 2 mm band in the region of tooth 21; and at an angle of 30° with a 3 mm band on the zygomatic implant, screw-retained with a torque of 20 N. The index was made with acrylic resin (Pattern Resin LS – GC) together with the impression transfer, using light condensation silicone (Optosil Xantopren - Kulzer) with the surgical guide. Three days after surgery, a post-operative session of soft tissue laser therapy was performed, the suture was removed and the implant supported hybrid denture with resin teeth and with bar cast in chrome/cobalt were delivered to the patient. The patient presented a hematoma on the lower eyelid on the right side. Hirudoid ointment was prescribed for application on the eyelid 3 times a day for 7 days. After delivery of the hybrid prosthesis, the patient had 7 consultations for evaluation, during which occlusal adjustments were made, radiographic follow-up and prophylaxis of the maxillary hybrid prosthesis and teeth in the maxillary arch were performed. In October 2010, the hybrid prosthesis was removed for relining of the spaces ere there had been peri-implant soft tissue resorption due to the extractions. In addition, 4 microunit prosthesis fixation screws were changed. After this date, the patient returned for preventive consultations another 7 times up to November 2014. In this period prophylaxes were performed. Teeth 11 and 12 of the hybrid prostheses, which had become loose, were replaced. As we observed that natural wear of the teeth had occurred due to bruxism, the patient was instructed to have a functional myorelaxant plate made and have a new prosthesis remade. In 2016, after contact by telephone, the patient said he had had the prosthesis remade by another dentist. In February 2019, the patient returned to our private clinic as tooth 11 of the hybrid prosthesis had become loose. Temporary repair of the prosthesis was made, and we proposed to the patient that he should have a new hybrid prosthesis with a personalized cast bar made to receive 12 ceramic teeth cemented to it. After the patient accepted the proposed treatment, the prosthesis was removed (Fig. 1), and the microunit abutment of the zygomatic implant was found to be loose. It was replaced with a torque of 20 N. The peri-implant tissues were affected by mucositis, due to the patient's poor oral hygiene and lack of periodic preventive control (Fig. 2). Prophylaxis was performed and an oral mouth wash (Periogard, Colgate) was prescribed. After this, the impression was taken for fabricating the occlusal orientation plane (Fig. 3). After the register and esthetic try-in of the resin teeth (Fig. 4), a personalized bar was fabricated (Fig. 5). After this stage and try-in of the bar, a try-in was made of 12 teeth made of wax, copying the same shapes as those of the resin teeth, approved by the patient, on the acrylic resin casings (Pattern Resin LS – GC) to enable a new esthetic try-in and for checking occlusion (Fig. 6) on the bar. The next step was to try-in the 12 ceramic crowns that were ready, and to make occlusal adjustments (Fig. 7). After approval by the patient, the crowns were cemented with Relyx (3 M) resin cement onto the chrome/cobalt metal structure, and prosthesis with artificial gingiva made of resin was acrylized (Fig. 8). The new hybrid prosthesis was delivered to the patient in April 2019 (Fig. 9). A panoramic radiograph was taken to verify the seating of the prosthesis on the microunit abutments (Fig. 10). The patient was given instructions about oral hygiene care and prevention.

## 3 Discussion

The placement of zygomatic implants is a challenging treatment [7]. In the clinical report presented, change in the surgical planning occurred due to the loss of the vestibular bone walls, leading to the surgeon option for placement of the zygomatic implant under local anesthesia and oral sedation. Only very experienced surgeons should perform this procedure in type of situation [1]. Over the course of ten years after beginning with the treatment, the patient attended preventive consultations, with a view to maintaining the health of peri-implant tissues and prosthetic system used. All patients who have zygomatic implants inserted must participate in a preventive program, which cooperates with achieving the success of this type of therapy over the course of years [1].

Right from the beginning of treatment, problems occurred, such as post-operative hematoma in the lower eyelid, loosening of teeth in the denture and of the zygomatic implant microunit abutment, considered common in this type of clinical approach [1,3,6]. The last prosthesis fabricated for the patient not only improved his self-esteem psychologically, because he was able to use dental floss between the teeth, but because it also prevented embarrassment in daily life contact with other persons, which made him extremely happy. A recent satisfaction survey recently conducted between two groups of patients revealed that this type of treatment improved their self-esteem and their general level of satisfaction was high. Oral hygiene was the only item that received a score below 7 in one of the groups, however, without significant difference [1]. In this clinical report, the patient was observed to experience difficulty with oral hygiene, right from the beginning of treatment. This occurred in relation to both cleaning the hybrid prosthesis and the natural teeth in the mandibular arch, which was lacking in various evaluations. Thus, it reinforced the idea that all patients must return for preventive consultation more frequently.

No sinusitis occurred in this patient. This is considered the major cause of complications, particularly when the trajectory of zygomatic implant lies within the maxillary sinus and there are threads along its entire surface [3,8]. In this clinical report, the zygomatic implant was of the internal hexagon type with threads on its entire body. At present, the use of implants with threads on the head and apex only is recommended, or even only on the apex, with a view to minimizing problems with sinusitis and dehiscence of the soft tissue over the head of the zygomatic implant. This is particularly the case when there is bone around the head, as occurs in the extra-maxillary technique [2,7,8].

The microunit abutment of the zygomatic implant was loose when the patient's old hybrid dental prosthesis was removed for taking the impression. This was in agreement with a recent study with finite element analysis, in which the highest stress received was localized on the posterior zygomatic implant and its microunit screw [7]. This showed the importance of periodic clinical consultations for checking the tightening of all the screws.

In spite of the small problems verified in this clinical report, treatment with zygomatic implants continues to be an excellent treatment option, with a high success rate, survival and patient satisfaction reported in the literature worldwide [1,6,7,10,11].

## 4 Conclusion

Therapy with zygomatic implants must be part of the treatment options presented to patients. Surgery may occur in the private clinic with local anesthesia and oral sedation when performed by experienced professionals. These treatments have shown high success and patient satisfaction rates, with improvement in quality of life. All patients must participate in a maintenance and oral hygiene program.

## Ethical approval

None declared.

## Funding

None.

## Author contribution

Study design: Paulo H. T. Almeida.

Data collection: Paulo H. T. Almeida.

Data interpretation: Paulo H. T. Almeida.

Manuscript preparation: Paulo H. T. Almeida.

Critical revision: Paulo H. T. Almeida, Sergio H. Cacciacane, Ayrton Arcazas Junior.

## Trial registry number

1. Name of the registry:
2. Unique Identifying number or registration ID:
3. Hyperlink to the registration (must be publicly accessible):

None declared.

## Guarantor

Paulo Henrique Teles de Almeida.

## Consent

Written informed consent was obtained from the patient for publication of this case report and accompanying images. A copy of the written consent is available for review by the Editor-in-Chief of this journal on request.

## Provenance and peer review

Not commissioned, externally peer reviewed.

## Declaration of competing interest

None declared.
